# Synthesis, characterization, in vitro antimicrobial, and U2OS tumoricidal activities of different coumarin derivatives

**DOI:** 10.1186/1752-153X-7-68

**Published:** 2013-04-15

**Authors:** Sadia Rehman, Muhammad Ikram, Robert J Baker, Muhammad Zubair, Effat Azad, Soyoung Min, Kashif Riaz, KH Mok, Saeed-Ur Rehman

**Affiliations:** 1 Institute of Chemical Sciences, University of Peshawar 25120, Peshawar, Pakistan; 2 Department of Chemistry, Sarhad University of Science and Information Technology, Peshawar, Pakistan; 3 School of Chemistry, University of Dublin, Trinity College Dublin 2, Dublin, Ireland; 4 Trinity Biomedical Sciences Institute (TBSI), School of Biochemistry and Immunology, Trinity College Dublin, Dublin 2, Ireland; 5 Department of Plant Pathology, University of Agriculture Faisalabad-Pakistan, Faisalabad, Pakistan

**Keywords:** Dicoumarols, Translactonized products, Antimicrobial activity, U2OS anticancer activity

## Abstract

**Background:**

Coumarin and its derivatives are biologically very active. It was found that the enhanced activities are dependent on the coumarin nucleus. Biological significance of these compounds include anti-bacterial, anti-thrombotic and vasodilatory, anti-mutagenic, lipoxygenase and cyclooxygenase inhibition, scavenging of reactive oxygen species, and anti-tumourigenic. Our interest in medicinal chemistry of dicoumarol compounds have been developed by keeping in view the importance of coumarins along with its derivatives in medicinal chemistry. All the synthesized compounds were fully characterized by spectroscopic and analytical techniques and were screened for antimicrobial and U2OS bone cancer activities.

**Results:**

4-hydroxycoumarin was derivatized by condensing with different aldehydes yielding the dicoumarol and translactonized products. Elemental analyses, ESI(+,−) MS, ^1^H and ^13^C{^1^H}-NMR, infrared spectroscopy and conductance studies were used to characterize the synthesized compounds which revealed the dicoumarol and dichromone structures for the compounds. The compounds were screened against U2OS cancerous cells and pathogenic micro organisms. The compounds with intermolecular H-bonding were found more active revealing a possible relationship among hydrogen bonding, cytotoxicity and antimicrobial activities.

**Conclusion:**

Coumarin based drugs can be designed for the possible treatment of U2OS leukemia.

## Background

### Medicinal application of coumarin derivatives

Coumarins are naturally occurring organic compounds [1,2] which belong to benzo-α-pyrones group of compounds. Coumarin derivatives are widely used in the field of medicines and drugs for many years [2-7] as for example coumarin and its derivatives exhibited pronounced anticancer and antimicrobial activities as revealed from literature. Coumarin derivatives have been applied for treatment of various cancerous diseases including malignant melanoma, leukemia, renal cell carcinoma, prostate and breast cancer [2-8]. When those compounds were applied in combination with radiotherapy and surgery as a chemotherapeutic agent provided best results, treating not only the cancer but also decreased the side effects of radio therapy [8].

Initially, the melanoma diagnosis involved just the surgical removal of primary lesion with the high risk of recurrence after five years. However, the problem has been minimized by the use of 4-hydroxy coumarin along with warfarin to maintain therapy and to inhibit the tumor spread [9]. In case of leukemia, prostate and breast cancer, cyclin D1 is released in an amount more than a normal levels and coumarin derivatives have also been found very effective antiproliferative agents by regulating the release of cyclin D1 [10-19].

### Types of sarcoma and its treatment

**Figure 1:**
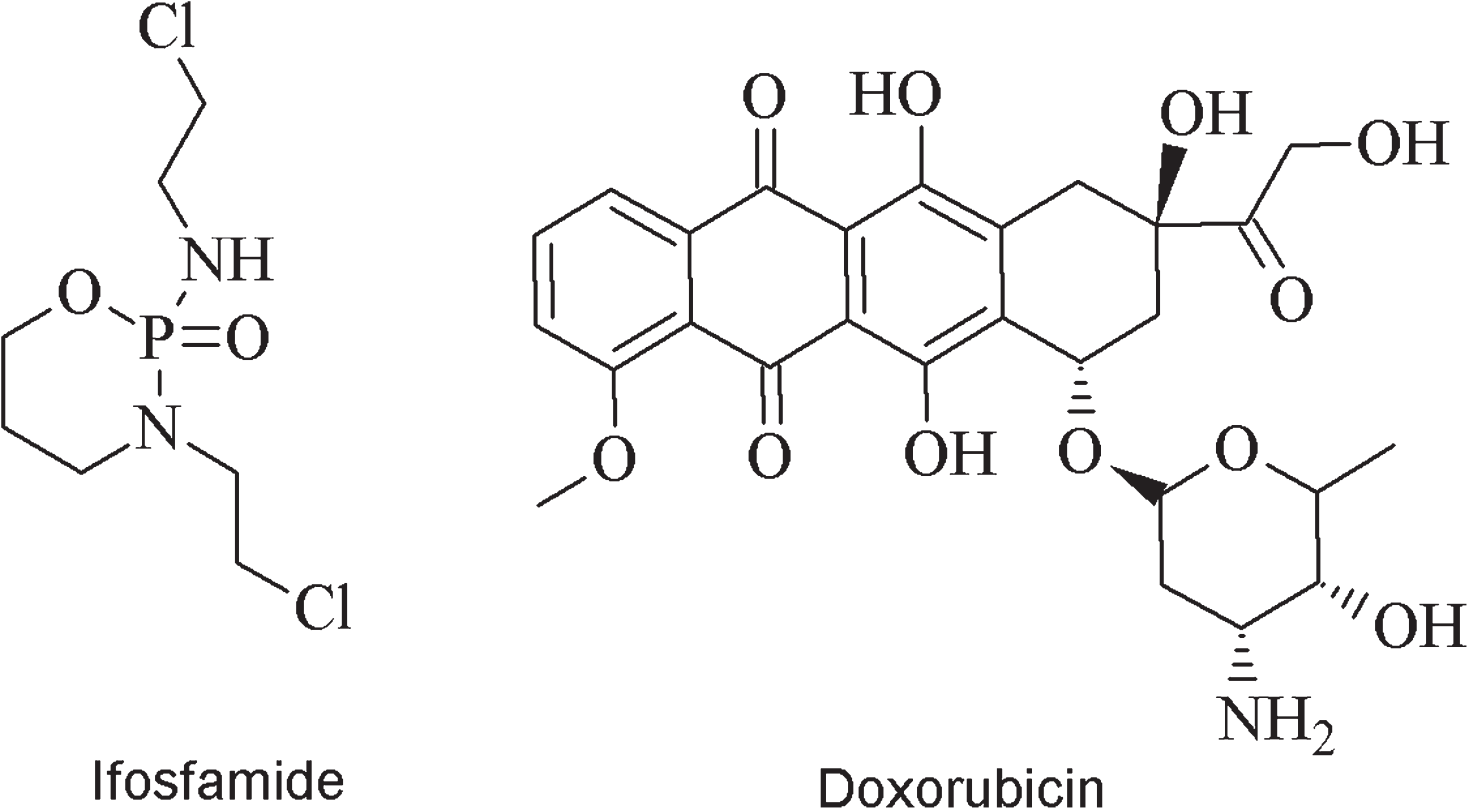
Drugs used for treating sarcoma.

There are different types of sarcomas including the synovial sarcoma, Ewing's sarcoma and osteosarcoma which are more often found in young adults and neoplasias such as leiomyosarcoma or liposarcoma are found in humans of more than 55 years age [20-22]. Synovial sarcoma is being treated using doxorubicin and ifosfamide [22]. The doxorubicin has been found to cause neutropenia, alopecia, dispigmentation, and reactivation of Hepatitis B, cardiomyopathy or even death. Therefore due to its very toxic nature it had been named as “Red Devil” [23-26] (Figure 1).

Similarly ifosfamide causes encephalopathy (brain dysfunction), a very serious drawback of the drug. Encephalopathy is actually caused by the production of toxic side products like acetaldehyde and chloral hydrate [25,26].

Coumarin and its derivatives are biologically very active. It was found that the enhanced activities are dependent on the coumarin nucleus [25,26]. Biological significance of these compounds include anti-bacterial [27], anti-thrombotic and vasodilatory [28], anti-mutagenic [29], lipoxygenase and cyclooxygenase inhibition [30,31], scavenging of reactive oxygen species, and anti-tumourigenic [32,33]. The fact that such compounds have medicinal applications, prompted many researchers to work in this field and several recent reviews summarize advances in this field [34-38].

**Scheme 1:**
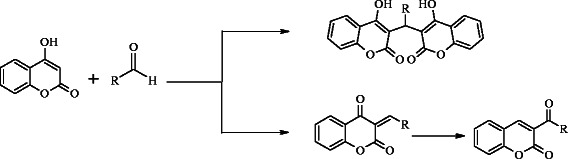
Synthesis of coumarine derivatives.

Our interest in medicinal chemistry of dicoumarol compounds have been developed by keeping in view the importance of coumarins along with its derivatives in medicinal chemistry. We envisioned the synthesis of dicoumarol compounds by reacting 4-hydroxy coumarin with various aldehydes (Scheme 1). All the synthesized compounds were fully characterized by spectroscopic and analytical techniques and were screened for antimicrobial and U2OS bone cancer activities.

## Result and discussion

### Spectral studies

4-hydroxy coumarin was condensed with different aldehydes in ethanol under reflux. All the compounds were characterized using different techniques including ^1^H, ^13^C{^1^H}-NMR, high resolution ESI-MS, elemental analysis, and infrared spectroscopic techniques. General reaction is given in Scheme 1.

It was observed that different substitution on the aldehyde center produced dicoumarol compounds except salicyldehyde and 2- hydroxynaphthaldehyde, the ortho hydroxy substituted aldehydes, yielded a mixture of translactonised and dicoumarol products. ^1^H-NMR revealed that all the dicoumarol compounds followed the Michael addition reaction. Condensation of benzaldehyde, 4-nitrobenzaldehyde, 4-chlorobenzaldehyde, 3-pyridinecarboxaldehyde, 3-indolecarboxaldehyde, 4-methoxybenzaldehyde, N,N-dimethyl-4-benzaldehyde and vaniline produced dicoumarol compounds which were unambiguously characterized and confirmed using spectroscopic and analytical techniques. Elemental analyses established the composition which was further confirmed by high resolution ESI(+,−) MS spectral analysis. The percent abundance coincides with the respective molecular ion peaks.

**Figure 2:**
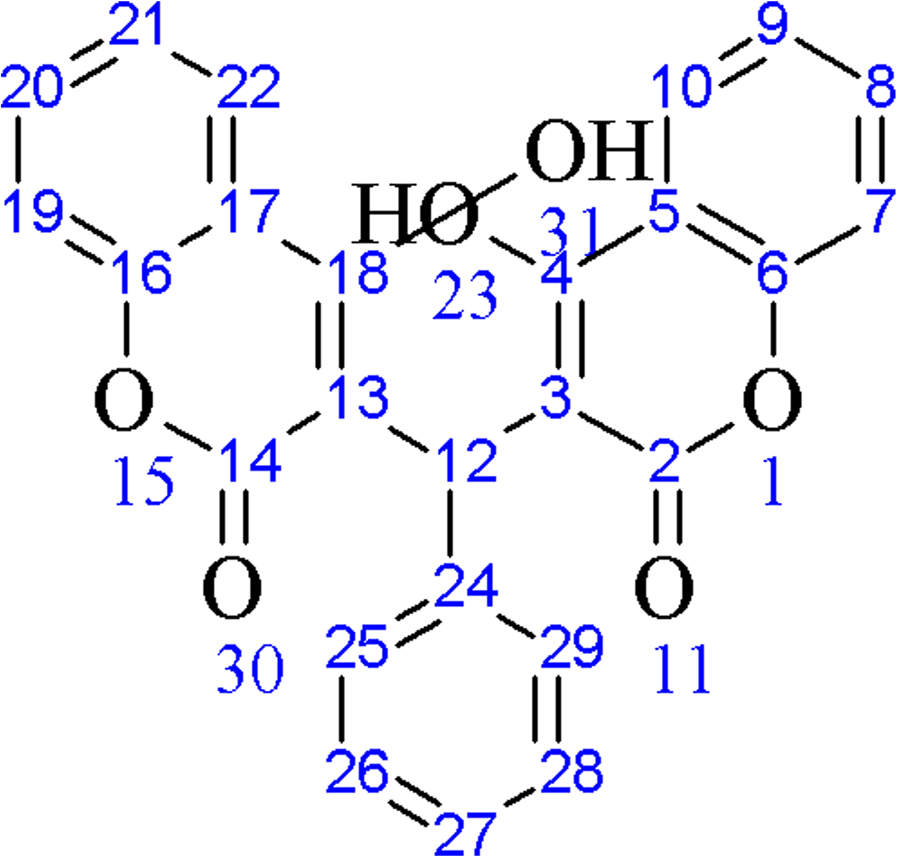
Atoms numbering used for NMR signals.

**Scheme 2:**
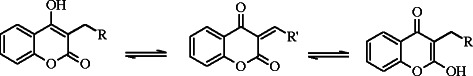
Tautomerism in coumarin structure.

^1^H and ^13^C{^1^H}-NMR were carried out for all the compounds using DMSO-*d*_*6*_. The NMR assignment for dicoumarols is based on Figure 2. The enolic protons were found to be involved in the formation of hydrogen bonding. A singlet at 10-12 ppm in different dicoumarol compounds were assigned to those enolic protons because they can be removed easily by adding deuterium oxide [20]. A singlet at 6 ppm was assigned to the methylene group of the aldehydes which connects both the moieties. This singlet can also be observed at about 9.8-12 ppm because of the possible tautomerism as may be seen in Scheme 2. Rest of the NMR spectra clearly coincides with their respective peaks for each proton.

**Scheme 3:**
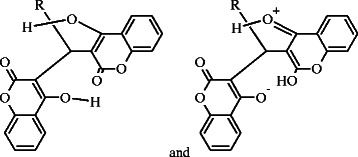
Hydrogen bonding in dicoumarol compounds.

The solid state ATR spectra were recorded for all the compounds in 400-4000 cm^−1^ spectral region. Since the IR spectra of all the compounds were quite similar therefore discussion is confined to the important vibrations only. The carbonyl stretching frequency varies from 1600-1730 cm^−1^ which may be due to the interaction of carbonyl group with the hydroxyl group to produce strong hydrogen bonding. Earlier studies revealed that carbonyl group involved in hydrogen bonding vibrates at lower frequency. The hydrogen bonding may either be intermolecular or intramolecular as shown in Scheme 3.

**Scheme 4:**
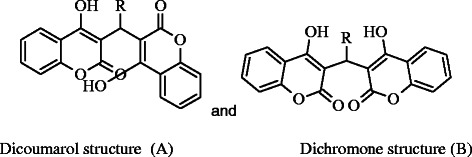
Dicoumarol and dicoumrone structures.

Compound **5**, **9** and **10** have carbonyl stretching frequencies at 1700-1730 cm^−1^ whereas **2**, **4**, **6** and **7** were observed to be vibrating around 1645-1680 cm^−1^, a dicoumarol structure (structure A) was assigned as shown in Scheme 4.

Compounds **1**, **3**, and **8** were observed to have carbonyl stretching frequency at 1600-1630 cm^−1^ which is around 30-60 cm^−1^ lower than the stretching frequency for **2**, **4**, **6** and **7,** a dichromone structure (structure B) was assigned as given in Scheme 4. Rest of the IR spectrum was assigned to their respective peaks [22].

Compound **5** and **10** were found to be a mixture of two products, a) dicoumarol compound and b) translactonized product (Scheme 1).

A weak band in the range of 1090-1120 cm^−1^ was assigned to the stretching frequency of C-O of lactone ring. Hydroxyl group is involved in hydrogen bond formation therefore the band is weakly observed. There is also a strong vibration around 1040 cm^−1^ in both the compounds which is assigned to the complementary band for the ketone carbonyl carbon.

**Figure 3:**
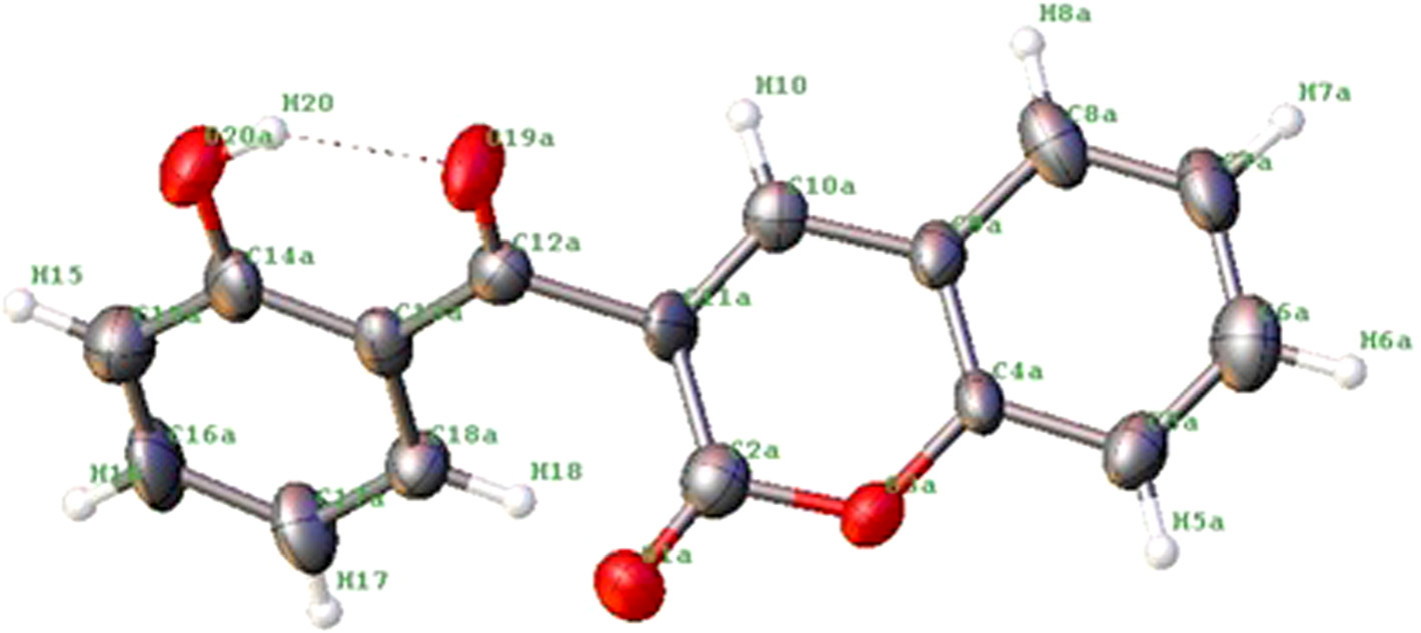
**Molecular Structure of 11 with labeled H-atoms.** (Numbering was used for NMR assignment of 11).

**Figure 4:**
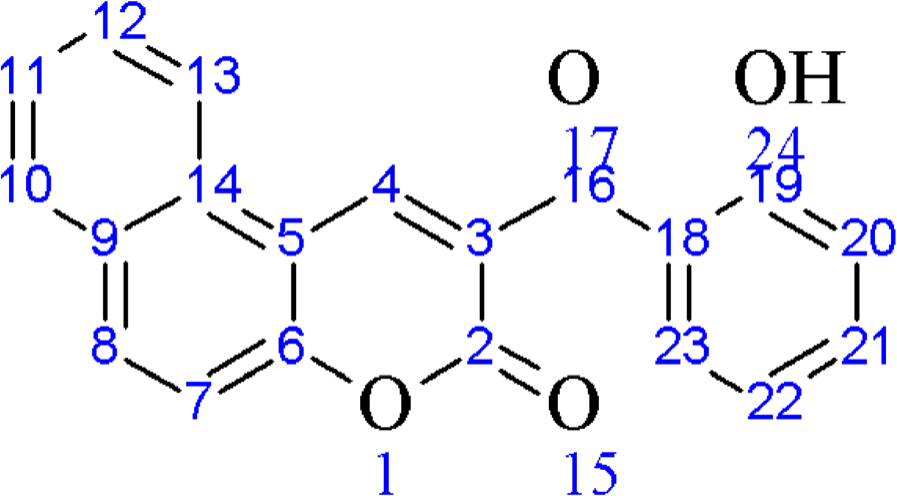
Labelled diagram of 12 for NMR assignment.

It was found that there is a type of competition between the two products which dominates the formation of translactonised product. The two products were separated by coloumn chromatography using alumina as stationary phase and methanol as eluent. Translactonised products being soluble in MeOH are collected first whereas the dicoumarol derivatives are collected by using methanol containing triethylamine (70% : 30%). It was found that 85-94% product is translactonised whereas the dicoumarol compound varies between 5-12% in abundance. ^1^H and ^13^C{^1^H}-NMR spectroscopy was performed to confirm the formation of those products. Figure 3 was used for assignment of resonances in **11** to their respective protons whereas Figure 4 was used for compound **12**. ^1^H-NMR for **11** show a singlet around 12.56 ppm which was assigned to the proton of phenolic OH group. Similarly, a singlet around 10.79 ppm was tentatively assigned to the H(10), a proton of methylene group of the coumarine ring. The protons at this position in compound **12** were observed around 10.70 ppm. The rest of the resonances for both compounds were assigned to the protons of aromatic ring system. The compounds were confirmed to be translactonized by studying the ^13^C{^1^H}-NMR which show peaks around 192 ppm, assigned to C=O group of ketone. Similarly the lactone C=O was assigned to the peak appearing around 181 ppm. These two peaks are identical in both the compounds. Rest of the spectrum was assigned to their respective carbon atoms. Infrared spectrum reveals absorption bands around 1714 cm^−1^ which was assigned to the C=O stretching frequency of the ketone. A weak band in the range of 1090-1120 cm^−1^ was assigned to the stretching frequency of C-O of lactone ring. The OH group is involved in H-bond formation and the band is weakly observed. There is also a strong vibration around 1040 cm^−1^ in both the compounds, which is assigned to the complementary band for the ketone C=O carbon.

### Crystal structure of 11

Compound **11** was crystalized out from MeOH solution of the product. The ORTEP plot of **11** is shown in Figure 3 and the crystal data in Table 1. A complete asymmetry was observed around the carbonyl carbon C(12a). This asymmetry can be seen by comparing the dihedral angle between the planes C(2a)-O(3a)-C(4a)-C(5a)-C(6a)-C(7a)-C(8a)-C(9a)-C(10a)-C(11a) and C(13a)-C(14a)-C(15a)-C(16a)-C(17 a)-C(18a) which was found to be 59.24^o^. The crystal packing diagram show that intermolecular hydrogen bonding between O(1a) and H(18) of the neighboring ring of 2.537A^o^ bond length is involved. Apart from it weak Van der Waals interactions may be seen between the rings C(2a)-O(3a)-C(4a)-C(5a)-C(6a)-C(7a)-C(8a)-C(9a)-C(10a)-C(11a) of two neighboring molecules. These interactions hold the rings by distance of 3.385A^o^ through π-π stacking. There is strong intramolecular hydrogen bonding of 1.823A^o^ length between O(19 a) and H(20).

**Table 1:** Crystal data and structure refinement for 11

	
Empirical formula	C_16_H_10_O_4_
Formula weight	266.24
Temperature/K	295(2)
Crystal system	orthorhombic
Space group	Pca2_1_
a/Å	25.668(2)
b/Å	4.0498(4)
c/Å	23.078(2)
α/°	90.00
β/°	90.00
γ/°	90.00
Volume/Å^3^	2399.0(3)
Z	8
ρ_calc_mg/mm^3^	1.474
m/mm^−1^	0.107
F(000)	1104.0
Crystal size/mm^3^	0.371 × 0.059 × 0.039
2Θ range for data collection	6.34 to 53.9°
Index ranges	−31 ≤ h ≤ 24, −2 ≤ k ≤ 4, −28 ≤ l ≤ 27
Reflections collected	6018
Independent reflections	2356 [R(int) = 0.093]
Data/restraints/parameters	2356/1/363
Goodness-of-fit on F^2^	1.040
Final R indexes [I>=2σ (I)]	R_1_ = 0.0874, wR_2_ = 0.1780
Final R indexes [all data]	R_1_ = 0.1558, wR_2_ = 0.2210
Largest diff. peak/hole / e Å^−3^	0.38/−0.26

### Antimicrobial activities

All the synthesized compounds were studied *In vitro* for their antimicrobial activities and the results are shown in Table 2. The table depicts that all the tested compounds are active against *Bacillus atrophaeus*, except compounds **5**, **6**, and **8** which showed moderate activities. Compounds **1**, **3**, **7** and **10** are more active against *Bacillus subtilis*, while all other compounds were moderately active. All the compounds were inactive against *Klebsiella pneumonia* and *Salmonella typhus* except some dicoumarols as may be seen in Table 2. Similarly moderate activities were observed against *Pseudomonas aeruginosa* and *Escherichia coli.* Interesting results were observed for compound **5** and **10** against *Candida albican and Agrobacterium tumefaciens* which were more active than the standard drug. Compounds **1**, **7**, **8** and **9** were also more active than the standard but their activities are less pronounced than compound **5** against *Candida albican*. Compound **6** also showed comparable activity against *Agrobacterium tumefaciens.* Except compound **2**, all the compounds were active against *Agrobacterium tumefaciens.* Compound **7** showed moderate activity *whereas* rest of the other compounds were found inactive against *Erwinia carotovora*.

**Table 2:** In Vitro antimicrobial activities of dicoumarols against different animal and plant pathogens

**Compounds**	***Bacillus atrophaeus***	***Bacillus subtilis***	***Klebsiella pneumoniae***	***Salmonella typhus***	***Pseudomonas aeruginosa***	***Escherichia coli***	***Staphylococcus aureus***	***Candida albican***	***Agrobacterium tumefaciens***	***Erwinia carotovora***
	***(mm)***	***(mm)***	***(mm)***	***(mm)***	***(mm)***	***(mm)***	***(mm)***	***(mm)***	***(mm)***	***(mm)***
1	25	22	---	---	20	14	16	22	22	15
2	12	09	09	06	09	---	16	12	10	10
3	26	22	---	---	16	---	09	15	20	---
4	17	17	---	---	18	12	__	12	16	11
5	15	12	---	11	15	10	13	30	20	---
6	13	15	---	---	18	---	13	19	27	13
7	21	22	---	12	18	12	11	20	24	17
8	16	20	11	---	20	09	15	20	20	---
9	21	21	12	10	19	19	09	21	20	15
10	22	25	---	---	15	12	12	---	28	11
11	23	22	15	12	21	21	10	21	20	15
12	15	15	---	10	10	15	14	30	21	---
Standard	25	26	29	42	36	38	34	16	15	26

**Gram positive bacteria:***Bacillus atrophaeus*, *Bacillus subtilis*, *Staphylococcus aureus*, standard used was erythromycin in 6 μM.

**Gram negative bacteria:***Klebsiella pneumoniae*, *Salmonella typhus*, *Pseudomonas aeruginosa*, *Escherichia coli, Agrobacterium tumefaciens*, *Erwinia carotovora,* standard used was ciprofloxacin in 6 μM.

**Fungal Strain:***Candida albican,* standard used was clothrimazol in 6 μM.

### Cytotoxic Activity

**Figure 5:**
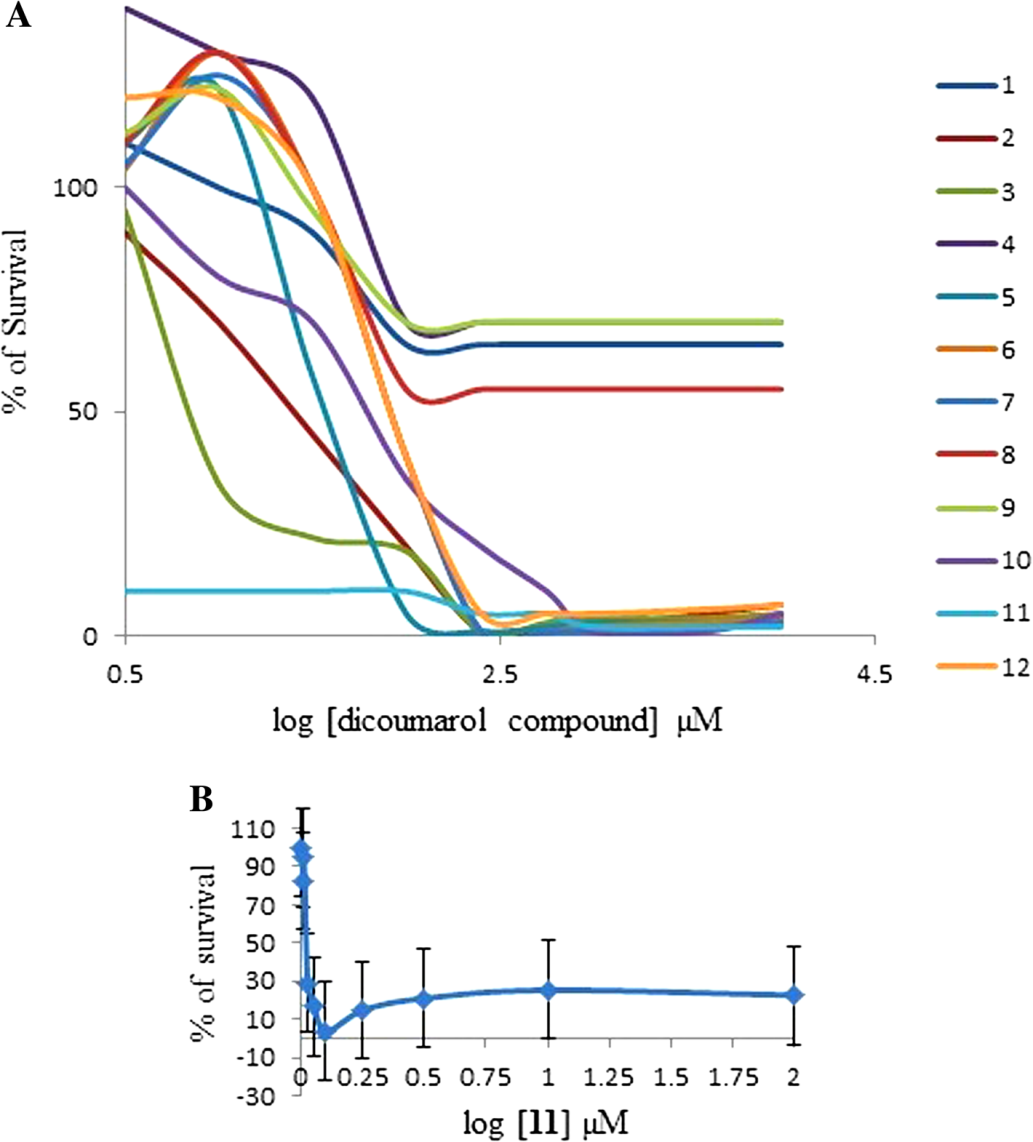
A) Cytotoxicity curve for the coumarin derivatives B) cytotoxicity curve for compound 11.

All the synthesized coumarin derivatives were subsequently studied for their cytotoxicity against U2OS osteoblast cancerous cells. The results are shown in Figure 5 which reveals that all the compounds have significant LD_50_ values except **3** which never dropped beyond 65% for its cytotoxicity. The compound **7** showed moderate LD_50_ value at 1.5 μM concentration while rest of the compounds exhibited LD_50_ at 2 μM concentration. Compound **10** showed LD_50_ value at very lower concentration unlike the others. The good results of compound **10** may be attributed to the presence of salicylic ring, an active part of majority of drugs, and also its activity may be due to the presence of hydroxyl group. Therefore it can be hypothesized from these observations that hydroxyl group and its orientation in a compound plays vital role in determining the cytotoxic activities of the active compounds.

Similarly the translactonized compounds **11** and **12** were also evaluated for their effectiveness against osteoblast cancerous cells. It can be revealed that both the compounds exhibited a well-behaved cytotoxicity curve with an LD_50_ value of 1.2 μM for **11** and 2 μM for **12**; Figure 1. The lower LD_50_ value for **11** as compared to **12** can be understood by looking into the structural features and solubilities. There is not much difference between the two compounds except the addition of extra hydrophobic phenyl ring. The added phenyl ring is making compound **12** harder to be soluble in coordinating low polarity solvents as compared to highly polar and strong coordinating solvents (like DMSO). Therefore it can be revealed that increasing fused ring system lowers the activity many folds by lowering the hydrophilic interaction.

Previously columbamine (COL), zoledronic acid, epigallocatechin gallate (EGCG) and an interleukin (IL)-1 receptor antagonist, huperine and monoclonal antibody were reported for their possible therapeutic application against U2OS sarcomyses [39-41]. Recently, Bao et al. [42] reported columbamine to be involved in effective inhibition of expression of MMP2 expressions at very low concentrations. But here the reported dicoumarol compounds like **3** and **10**, translactonised products like **11** and **12** demonstrated effective inhibition of U2OS sarcoma as compared to the COL. The same mechanistic approach for the effectiveness of the coumarin based tumoricidal agents can be assigned which involves the suppression of neovascularization, adhesion, migration, and invasion. The cytotoxic concentrations are very low as compared to the reported drugs therefore U2OS tumoricidal agents can be designed using the **3**, **10**, **11** and **12** coumarin based compounds.

## Experimental

### Materials and methods

All chemicals, buffers and solvents used were of analytical grade. Benzaldehyde, 4-nitrobenzaldehyde, 4-chlorobenzaldehyde, and N,N-dimethyl-4-benzaldehyde were obtained from Fluka whereas 3-pyridinecarboxaldehyde, 3-indolecarboxaldehyde, 4-methoxybenzaldehyde, vaniline, 2-hydroxynaphthaldehyde and salicyldehyde were obtained from Sigma Aldrich and were used as such without further purification. Solvents were obtained from local suppliers of Sigma Aldrich, Merck and Fluka and were distilled at least twice before use. Unless otherwise stated, all reactions were carried out under dinitrogen atmosphere.

### Instrumentation

Elemental analyses were carried out on Varian Elementar II. IR spectra were recorded using Shimadzo FTIR Spectrophotometer Prestige-21. ^1^H-NMR were measured with Bruker DPX 400 MHz (400.23 MHz) whereas, ^13^C{^1^H}NMR were recorded on Bruker AV 400 MHz (150.9 MHz) spectrometers in , (CD_3_)_2_OS at room temperature. Chemical shifts are reported in ppm and standardized by observing signals for residual protons. Molar conductance of the solutions of the metal complexes was determined with a conductivity meter type HI-8333. All measurements were carried out at room temperature with freshly prepared solutions. Mass spectra were recorded on a LCT Orthogonal Acceleration TOF Electrospray mass spectrometer.

### Crystal Structure Determination

Single crystal analyses were carried out using suitable single crystals for X-ray structural analyses of **11** was mounted on a glass fiber, and the respective data were collected on oxford diffractometer (graphite-monochromated Mo Kα radiation, λ = 0.71073 Å) at 108(2) K. The structures were solved with the olex2.solve [43] structure solution program using Charge Flipping and refined with the olex2.refine [Bourhis LJ, Dolomanov OV, Gildea RJ, Howard JAK, Puschmann H: *Olex2.refin.* 2011. in preparation] refinement package using Gauss-Newton minimization. Crystallographic details are given in the Additional file 1 CCDC 888786 (1) data can be obtained free of charge from the Cambridge Crystallographic Data Centre via http://www.ccdc.cam.ac.uk/data_request/cif.

### Antimicrobial activity

About 2.8 g/L nutrient agar and nutrient broth were prepared in deionized water and kept in autoclave set at 1.5 Pounds pressure for about 15 min. Under inert atmosphere the nutrient agar media were poured aseptically into sterilized petri dishes in laminar flow. The petri dishes were kept for about 24 hrs. at 37°C in inverted position. Bacterial cultures were adjusted to 0.5 McFarland turbidity standards and Candida albican was adjusted to 108 cfu/ml. Sterile filter paper of diameter 6 mm was used for bacterial strains whereas its thickness ranged upto 13 mm for fungal strains. These filter papers were in the form of discs and were seeded with 0.5 McFarland and 106 cfu/ml cultures of bacteria and fungi respectively. Solutions (0.5 mM) of the synthesized compounds were applied to the prepared discs and incubated for 18 hr at 37°C. Subsequent measurements of the zone of activity were carried out [21].

### Cytotoxic activity

The human osteosarcoma cell line U2OS was used for testing the tumoricidal activities of all coumarin compounds. The cells were cultured in DMEM+ GlutaMAXTM−1 with added 1% penicillin and streptomycin and 10% heat-inactivated fetal bovine serum. For adherent cells, trypsin-EDTA was used for detachment. The cells were washed in Dulbecco’s phosphate buffered saline (DPBS), harvested by centrifugation (1000 rpm, 5 min), and resuspended in DMEM. The cells were seeded into 96-well plates at a density of 5 × 10^3^ cells / well, and incubated at 37°C in 5% CO_2_ atmosphere for 24 hours before being treated with the compounds. The compounds were initially dissolved at a stock concentration of 10 mM in DMSO and added to wells to a final concentration between 5.0 and 200 μM. The plates were then incubated at 37°C for a further 24 hours for treatment. Alamar blue (Invitrogen) was used to test the viability of the cells (10 μl per well). Plates in triplicate were incubated for 4 hours at 37°C protected from direct light, then read at 590 nm using an excitation wavelength of 544 nm in a fluorescence plate reader (Spectra Max Gemini). Wells containing medium and distilled water-only served as blank controls, while the viability of the treated cells was taken as a percentage compared to wells with untreated cells. The LD_50_ value of each compound was estimated by fitting the correlation between cell viability and compound concentration.

### Synthesis of dicoumarol ligands

The dicoumarol ligands were synthesized by the reported procedure with slight modifications [22], 25 mmol of the aldehydes were added to the 50 mmol stirred ethanolic solution of 4-hydroxycoumarin and the mixture was refluxed for 3 hr at 120°C. After cooling the reaction mixture, solid white powder of the ligands were isolated, washed several times with copious 10% ethanolic n-hexane solution. The product was purified by dissolving in methanolic solution containing small volume of triethylamine. The process repeated two times to get pure recrystalised dicoumarol ligand. The formation of dicoumarol ligand may be seen in Scheme 1.

#### 3.6.1 3,3'-(phenylmethanediyl)bis(4-hydroxy-2H-chromen-2-one) (**1**)

IR: 3317(s), 3244(s), 3010(s), 1600(s), 1537(w), 1485(w), 1463(w), 1446(w), 1379(w), 1327(s), 1247(s), 1188(w), 1126(w), 1072(w), 1010(w), 831(s), 761(s), 711(s), 665(s) cm^−1^, ^1^H-NMR (400.23 MHz, (CD_3_)_2_OS, 303 k): *δ* = 6.31 (s, 1H, C12), 7.05-7.95 (m, H-aromatic), 10.01 (s, 2H, OH); ^13^C{^1^H}-NMR (150.9 MHz, (CD_3_)_2_OS,) (δ, ppm): 35.88 (C12); 102.66-131.74, (Carom); 152.44 (C4,C18), 165.63 (C2,C14), Elemental Analysis (C_25_H_16_O_6_), Calc. C: 72.81%, H: 3.91%, Exp. C: 72.80%, H: 4.10%, ESI-MS: *m/z* (%) 435.0845 (100%) [C_25_H_16_O_6_+Na]^−^.

#### 3.6.2 3,3'-(pyridin-3-ylmethanediyl)bis(4-hydroxy-2H-chromen-2-one) (**2**)

IR: 3500(bd), 3061(w), 2910(s), 2131(bd), 1681(s), 1610(s), 1531(s), 1456(s), 1408(s), 1348(w), 1276(s), 1253(w), 1178(s), 1105(s), 1049(s), 1004(s), 941(s), 900(w), 848(w), 806(s), 748(s), 677(s), 626(s), 605(s), 551(s) cm^−1^, ^1^H-NMR (400.23 MHz, (CD_3_)_2_OS, 303 k): *δ* = 6.40 (s, 1H, C12), 7.25-8.71 (m, H-aromatic), 10.13 (s, 1H, -OH), ^13^C{^1^H}-NMR (150.9 MHz, (CD_3_)_2_OS,) (δ, ppm): 101.71 (CH, C12), 115.74-144.62(Carom), 152.64 (C4,C18), 164.02 (C2,C14), Elemental Analysis (C_24_H_15_NO_6_), Calc. C: 69.73%, H: 3.66%, N: 3.39%, Exp. C: 66.80%, H: 3.10%, N: 3.89%, EI-MS: *m/z* (%) 412.0821 (100%) [C_24_H_15_NO_6_-H]^−^.

#### 3.6.3 3,3'-(1H-indole-3-ylmethanediyl)bis(4-hydroxy-2H-chromen-2-one) (**3**)

IR: 3350(bd), 2980(w), 2740(bd), 1614(s), 1516(s), 1470(s), 1338(s), 1294(s), 1242(s), 1193(s), 1116(s), 952(s), 831(s), 756(s), 734(s), 638(s) cm^−1^, ^1^H-NMR (400.23 MHz, (CD_3_)_2_OS, 303 k): *δ* = 5.60 (s, 1H, C12), 7.19-8.30 (m, H-aromatic), 9.93 (s, 1H, NH), 12.16 (s, 1H, OH), ^13^C{^1^H}-NMR (150.9 MHz, (CD_3_)_2_OS,) (δ, ppm): 90.85 (CH, C12), 112.34-138.73 (C, C aromatic), 153.53 (C4,C18), 162.03 (indole), 165.63 (CH, indole), 185.19 (C2,C14), Elemental Analysis (C_27_H_17_NO_68_), Calc. C: 71.84%, H: 3.80%, N: 3.10%, Exp. C: 66.80%, H: 3.31%, N: 3.09%, EI-MS: *m/z* (%) 451.0923 (100%) [C_27_H_17_NO_6_^+^].

#### 3.6.4 3,3'-[(4-methoxyphenyl)methanediyl]bis(4-hydroxy-2H-chromen-2-one) (**4**)

IR: 2800(w), 1650(s), 1602(s), 1558(s), 1506(s), 1448(w), 1352(s), 1305(s), 1257(s), 1176(s), 1093(s), 1053(s), 960(s), 904(s), 767(s), 673(s) cm^−1^, ^1^H-NMR (400.23 MHz, (CD_3_)_2_OS, 303 k): *δ* = 3.72 (s, 3H, OCH3); 6.23 (s, 1H, C12), 6.75-7.85 (m, H-aromatic), 10.01 (s, 1H, OH); ^13^C{^1^H}-NMR (150.9 MHz, (CD_3_)_2_OS,) (δ, ppm): 54.87 (CH^3^, -OCH^3^), 103.78 (CH, C12), 112.12-131.33 (C, C-aromatic), 152.18 (C4,C18), 169.70 (C2,C14), Elemental Analysis (C_26_H_18_O_7_), Calc. C: 70.58%, H: 4.10%, Exp. C: 70.80%, H: 4.92%, EI-MS: *m/z* (%) 441.0974 (100%) [C_26_H_17_O_7_-H^+^].

#### 3.6.5 3,3'-[(2-hydroxy-1,2-dihydronaphthalen-1-yl)bis(4-hydroxy-2H-chromen-2-one) (**5**)

IR: 3066(w), 1705(s), 1629(s), 1566(s), 1487(s), 1394(s), 1286(s), 1249(s), 1205(s), 1149(s), 1091(s), 1004(w), 927(s), 873(s), 817(s), 744(s), 677(s), 617(s) cm^−1^, ^1^H-NMR (400.23 MHz, DMSO-*d*^*6*^, 303 k): *δ* = 5.60 (s, 1H, C12), 6.94-8.64 (m, H-aromatic), 10.70 14 (s, 1H, Ar-OH), ppm, ^13^C{^1^H}-NMR (150.9 MHz, DMSO-*d*^*6*^, 303 k): *δ* = 192 (C2,C14)ppm, 159 (Ar-OH), 158 (C4,C18), 138.8-123 (Aromatic carbons), 98.23 (CH, C12), ppm, Elemental Analysis (C_29_H_20_O_7_), Calc. C: 72.49%, H: 4.20%, Exp. C: 72.60%, H: 4.12, EI-MS: *m/z* (%) 477.0974 (5%) [C_29_H_17_O_7_-H^+^], 316.0670 (30%) [C_20_H_12_O_4_] ^+^, 315.0496 (100%) [C_20_H_12_O_4_-H]^+^.

#### 3.6.6 3,3'-[(4-nitrophenyl)methanediyl]bis(4-hydroxy-2H-chromen-2-one) (**6**)

IR: 3200(bd), 2750(w), 1645(s), 1600(s), 1523(s), 1454(s), 1342(s), 1309(s), 1265(s), 1184(s), 1099(s), 1029(s), 960(s), 912(s), 813(s), 792(s), 675(s) cm^−1^, ^1^H-NMR (400.23 MHz, (CD_3_)_2_OS, 303 k): *δ* = 6.34 (s, 1H, C12), 7.23-8.54 (m, H-aromatic), 10.14 (s, 1H, Ar-OH), ^13^C{^1^H}-NMR (150.9 MHz, (CD_3_)_2_OS, 303 k), *δ* = 97.61 (CH, C12), 115.22-147 (C, C aromatic), 152., 27 (C4,C18), 164.49 (C2,C14)ppm, Elemental Analysis (C_25_H_15_NO_8_), Calc. C: 65.65%, H: 3.31%, N: 3.06%, Exp. C: 66.80%, H: 3.10%, N: 3.89%, EI-MS: *m/z* (%) 456.0719 (100%) [C_25_H_15_NO_8_-H]^+^.

#### 3.6.7 3,3'-[(4-chlorophenyl)methanediyl]bis(4-hydroxy-2H-chromen-2-one) (7)

IR: 2740(w), 1662(s), 1602(s), 1558(s), 1489(s), 1454(w), 1350(s), 1307(s), 1219(w), 1093(s), 1016(s), 954(w), 906(s), 819(s), 763(s), 673(s) cm^−1^, ^1^H-NMR (400.23 MHz, (CD_3_)_2_OS, 303 k): *δ* = 5.61 (s, 1H, C12), 6.28-7.93 (m, H-aromatic), ^13^C{^1^H}-NMR (150.9 MHz, (CD_3_)_2_OS, 303 k), δ = 103.50 (CH, C12), 115.97-140.26 (C, aromatic), 152.21 (C4,C18), 164.22 (C2,C14)ppm, Elemental Analysis (C_25_H_15_ClO_6_), Calc. C: 67.20%, H: 3.38%, Exp. C: 67.80%, H: 3.31%, EI-MS: *m/z* (%) 445.0479 (100%) [C_25_H_14_O_6_Cl−H]^−^.

#### 3.6.8 3,3'-[(4-N,N-dimethylaminophenyl)methanediyl]bis(4-hydroxy-2H-chromen-2-one) (**8**)

IR: 3356(bd), 2700(w), 2600(bd), 1614(s), 1598(s), 1548(s), 1516(w), 1342(s), 1323(s), 1292(s), 1273(s), 1242(s), 1188(s), 952(s), 935(s), 829(s), 756(s), 704(s), 682(s), 667((s), 651(s) cm^−1^, ^1^H-NMR (400.23 MHz, (CD_3_)_2_OS, 303 k): *δ* = 2.50 (s, 6H, NCH3), 5.60 (s, 1H, C12), 7.32-7.96 (m, H-aromatic), ^13^C{^1^H}-NMR (150.9 MHz, (CD_3_)_2_OS, 303 k), δ = 56.23 (CH3, NCH3), 91.05(CH, C12), 115.82-153.72 (C, C aromatic), 161.93 (C4,C18), 165.36 (C2,C14)ppm, Elemental Analysis (C_27_H_21_NO_6_), Calc. C: 71.20%, H: 4.65%, N: 3.08%, Exp. C: 71.83%, H: 4.60%, N: 3.98%, (EI-MS: *m/z* (%) 456.0919 (100%) [C_27_H_21_NO_6_+H]^−^.

#### 3.6.9 3,3'-[(3-methoxy-4-hydroxyphenyl)methanediyl]bis(4-hydroxy-2H-chromen-2-one) (**9**)

IR analysis: 3063 (m), 1703 (s), 1622 (m), 1599 (m), 1567 (s), 1512 (m), 1486 (s), 1439 (m), 1395 (s), 1339 (w), 1325 (m), 1305 (s), 1287 (s), 1249 (s), 1205 (s), 1180 (m), 1149 (w), 1092 (s), 1051 (m), 1033 (m), 995 (s), 973 (m), 949 (s), 926 (s), 873 (s), 854 (m), 830 (m), 817 (s), 805 (s), 794 (m), 782 (m), 744 (s), 716 (w), 676 (s), 665 (s) cm^−1^, ^1^H-NMR (400.23 MHz, (CD_3_)_2_OS, 303 k): *δ* = 2.38 (s, 3H, OCH^3^), 5.61(s, 1H, C12), 6.20-7.87 (m, H-aromatic), 9.76 (s, 1H, -OH), ^13^C{^1^H}-NMR (150.9 MHz, (CD_3_)_2_OS, 303 k), δ = 55.72 (CH3, OCH3), 103.98 (CH, C12), 111.65-147.21 (C & CH, C aromatic), 152.23 (C4,C18), 164.77 (C2,C14)ppm, Elemental Analysis (C_26_H_18_O_8_), Calc. C: 68.12%, H: 3.96%, Exp. C: 68.23%, H: 4.10%, EI-MS: *m/z* (%) 457.0923 (100%) [C_26_H_17_O_8_ −H] ^−^.

#### 3.6.10 3,3'-[(4-hydroxyphenyl)methanediyl]bis(4-hydroxy-2H-chromen-2-one) (**10**)

IR analysis: 3400 (bd), 3070 (w), 1714 (s), 1605 (s), 1588 (s), 1561 (s), 1487 (s), 1453 (m), 1389 (m), 1342 (s), 1311 (w), 1275 (s), 1240 (m), 1212 (s), 1171 (m), 1106 (w), 1037 (s), 956 (s), 939 (s), 919 (m), 862 (m), 809 (s), 743 (s), 674 (m) cm^−1^, ^1^H-NMR (400.23 MHz, DMSO-*d*^*6*^, 303 k): *δ* = 5.78 (s, 1H, C12), 7.51-10.79(m, H-aromatic), 12.56(s, 1H, -OH), ^13^C{^1^H}-NMR (150.9 MHz, DMSO-*d*^*6*^, 303 k): *δ* = 98.23 (CH, C12), 112-139 (C & CH, C aromatic), 158(C, C14a), 161(C, Ar-OH), 181(C2,C14), ppm. Elemental Analysis (C_25_H_18_O_7_), Calc. C: 69.76%, H: 4.22%, Exp. C: 69.88%, H: 4.60%, EI-MS: *m/z* (%) 427.0818 (10%) [C_25_H_15_O_7_-H^+^], 265.0429 (80%) [C_26_H_10_O_4_] ^+^.

### 3.7 Synthesis of 3-(2-hydroxybenzoyl)-2*H*-chromen-2-one (11) and 3-(2-hydroxybenzoyl)-2*H*-benzo[*g*]chromen-2-one (12)

A mixture of 4.86 g (0.03 mol) 4-hydroxy-2*H*-chromen-2-one and corresponding weights required for each aldehydes for 0.02 mol were mixed in 25 ml distilled and dried ethanol. This mixture was refluxed for 2-3 hrs depending upon the formation of solid products for each aldehyde. After the formation of solid yellow products for both **11** and **12**, it was filtered and washed three times with 2:1 mixture of methanol containing water and then with n-hexane. **11** was recrystallized from methanol by keeping the solution at room temperature for 24 hr.

#### 3.7.1 3-(2-hydroxybenzoyl)-2H-chromen-2-one (11)

IR analysis: 3400(bd), 3070(w), 1714(s), 1605(s), 1588(s), 1561(s), 1487(s), 1453(m), 1389(m), 1342(s), 1311(w), 1275(s), 1240(m), 1212(s), 1171(m), 1106(w), 1037(s), 956(s), 939(s), 919(m), 862(m), 809(s), 743(s), 674(m) cm^-1^, ^1^H-NMR (400.23 MHz, DMSO-*d*^6^, 303 k): *δ* = 7.51 (t, 1H, H8a), 7.66(d, 1H, H7a), 7.76 (m, 1H, H6a), 7.83(d, 1H, H16a), 8.11(d, 1H, H17a), 8.31(d, 1H, H5a), 8.64(d, 1H, H15), 9.11(s, 1H, H18), 10.79(s, 1H, H10), 12.56(s, 1H, -OH), ^13^C{^1^H}-NMR (150.9 MHz, DMSO-d^6^, 303 k): *δ* = 192(C, C12a), 181(C, C2), 165 (CH, C10a), 161(CH, C18a) 158(C, C14a), 153(C, C4a), 139(CH, C15a), 135(CH, C5a), 132(CH, C17a), 131(C, C13a), 126(C, C11a), 122(CH, C16a), 119(CH, C8a), 116(CH, C6a) 112(C, C9a), 91(CH, C7a) ppm, Elemental analysis: Exp. C: 72.23%, H: 3.67%, Calc. C: 72.18%, H: 3.79%, Mass spectra M+(%): 289.0473 (100%)[C16H10O4+Na]+.

#### 3.7.2 2-(2-hydroxybenzoyl)-3*H*-benzo[*f*]chromen-3-one (2)

IR analysis: 3063(m), 1703(s), 1622(m), 1599(m), 1567(s), 1512(m), 1486(s), 1439(m), 1395(s), 1339(w), 1325(m), 1305(s), 1287(s), 1249(s), 1205(s), 1180(m), 1149(w), 1092(s), 1051(m), 1033(m), 995(s), 973(m), 949(s), 926(s), 873(s), 854(m), 830(m), 817(s), 805(s), 794(m), 782(m), 744(s), 716(w), 676(s), 665(s) cm^-1^, ^1^H-NMR (400.23 MHz, DMSO-d^6^, 303 k): *δ* = 5.60(s, 1H, H4), 6.94 (m, 1H, H11 & H12), 7.36 (m, 1H, H13), 7.51 (m, 1H, H10), 7.65 (d, 1H, H8), 7.76 (d, 1H, H7), 7.83 (d, 1H, H21), 8.10 (d, 1H, H22), 8.31 (d, 1H, H23), 8.64 (d, 1H, H20), 10.70 (s, 1H, H2) ppm, ^13^C{^1^H}-NMR (150.9 MHz, DMSO-d^6^, 303 k): *δ* = 192(C, C16), 181 (C, C2), 165.7 (CH, C10), 161.8 (CH, C23), 161.5 (C, C19), 159 (C, C6), 158 (CH, C4), 138.8 (CH, C13), 135 (CH, C20), 134.7 (C, C5), 132.7 (C, C9), 131 (CH, C22), 129.9 (CH, C21), 128.8 (CH, C8), 127.8 (CH, C7), 126 (C, C3), 123.8 (CH, C11), 123 (CH, C12) ppm, Elemental analysis: Exp. C: 75.28%, H: 3.41%, Calc. C: 75.94%, H: 3.82%, EI-MS+ (%): 339.0633 (70%) [C20H12O4+ Na]+.

## Conclusion

Derivatizatin of 4-hydroxy coumarin have been carried out with different aldehydes. The compounds were characterized by various spectro-analytical techniques and it was confirmed that mixtures of dicoumarol and translactonized products were obtained for compounds **5** and **10**, compounds **1**, **3** and **8** bear dicoumarol and compound **2**, **4**, **6** and **7** exhibited dichromone structures. Apart from it **11** was recrystallized and studied for single crystal diffraction. The compound show *Pca2*_*1*_ space group and is connected by intermolecular hydrogen bonding in the crystal lattice. The in vitro cytotoxic screening of all the compounds and antimicrobial activities against different pathogenic microorganisms were carried out. The compounds **1**, **5**, **7** and **10** were more active against microbes than the standard drug used. In case of anticancer studies, the compound **10** and **11** have been found most active against U2OS cancerous cells than the previously reported tumorcidal drugs. Therefore the effectiveness of these compounds can be utilized to design significant anticancer agents.

## Competing interests

The authors declare that they have no competing interests.

## Authors’ contribution

SR carried out the synthesis of all the compounds. She also helped in solving the crystal data of the mentioned compound. MI helped a lot in write up and technical aspects of the work. KR helped in antimicrobial activities. RJB and MZ were involved in getting the spectral analyses of the synthesized compounds. EA, SM and KHM were the actual contributors for carrying out the bone cancer studies. SUR acted as supervisor for this work. All authors read and approved the final manuscript.

## Supplementary Material

Additional file 1: Figure S1 1H and ^1^H{^13^C}NMR of all the compounds. X-ray diffraction data. Click here for file
