# Supplementary material for: Synthesis, characterization, in vitro antimicrobial, and U2OS tumoricidal activities of different coumarin derivatives
Source: Chem Cent J. 2013 Apr 15;7:68. doi: 10.1186/1752-153X-7-68 (PMC3668295; doi:10.1186/1752-153X-7-68)
Supplement: Additional file 1: Figure S1 — 1H and 1H{13C}NMR of all the compounds. X-ray diffraction data. [file 1752-153X-7-68-S1.docx]

Figure 1. ^1^H{^13^C}NMR of **1**

Figure 2. ^13^C-NMR of **1**

Figure 3. ^1^H{^13^C}NMR of **2**

Figure 4. ^13^C-NMR of **2**

Figure 5. ^1^H{^13^C}NMR of **3**

Figure 6. ^13^C-NMR of **3**

Figure 7. ^1^H{^13^C}NMR of **4**

Figure 8. ^13^C-NMR of **4**

Figure 9. ^1^H{^13^C}NMR of **5**

Figure 10. ^13^C-NMR of **5**

Figure 11. ^1^H{^13^C}NMR of **6**

Figure 12. ^13^C-NMR of **6**

Figure 13. ^1^H{^13^C}NMR of **7**

Figure 14. ^13^C-NMR of **7**

Figure 15. ^1^H{^13^C}NMR of **8**

Figure 16. ^13^C-NMR of **8**

Figure 17. ^1^H{^13^C}NMR of **9**

Figure 18. ^13^C-NMR of **9**

Figure 19. ^1^H{^13^C}NMR of **10**

Figure 20. ^13^C-NMR of **10**

Figure 21. ^1^H{^13^C}NMR of **11**

Figure 22. ^13^C-NMR of **11**

Figure 23. ^1^H{^13^C}NMR of **12**

Figure 24. ^13^C-NMR of **12**
